# Selections

**Published:** 1862-04

**Authors:** 


### SELECTIONS.

METROPOLITAN HEALTH BILL.—"Preventive medicine will effect infinitely more for mankind than all the drugs which have yet been discovered, and all the curative skill which has ever been exerted for the alleviation of disease."—*John Simons, Health Officer of London.*

THE BILL IN ABSTRACT.—Section 1 provides for the appointment, by the Governor and the Senate, of seven citizens of the district, three of whom shall be practicing physicians, and who with the Health Officer, the Mayors of New York and Brooklyn, and Chairman of the Supervisors of Richmond County, shall be called the *Metropolitan Board of Health.* The members first appointed shall serve from one to seven years, and one shall afterwards be appointed annually to serve seven years.

§ 2.—To said Board of Health are given full power and authority to administer all the laws relating to the public health, interments, registration of births, marriages and deaths; and also to determine and regulate the diseases and vessels subject to Quarantine, and the anchorage of infected vessels; also to enforce the laws prohibiting or regulating the sale of poisonous, adulterated or unwholesome drugs, medicine and food.

§ 3.—Gives to the Board of Health the power to enforce the cleanliness of the streets and public places.

§ 4.—Gives control of domicilary nuisances, houses unfit for dwellings are prohibited, and landlords are required to keep their premises in proper condition, and penalties for neglect are prescribed.

§ 5.—All institutions, supported wholly or in part at public expense, are required to make such reports to the Board of Health as may enable them to ascertain the sanitary condition of any part of the district. The appointed members of the Board are subject to removal in the same manner as sheriffs. "A practicing physician of skill and experience" shall be appointed chief executive officer, and an Inspector of Health for the County of Kings, of like qualifications.

§§ 6 and 7 Fix the emoluments of the appointed members of the Board, and the mode of defraying the expenses incurred for the administration of the Act.

§§ 8 and 9 Repeal all inconsistent laws, and direct the immediate organization of the Board of Health.

"We believe that within this city, every year, thousands of lives are lost, which might be saved; that tens of thousands of cases of sickness occur, which might be prevented; that a vast amount of unnecessarily impaired health and physical debility exists among those not actually confined by sickness; that these *preventable* evils cause an enormous expenditure and loss of money, and impose upon the people unnumbered and immeasurable calamities, pecuniary, social, physical, mental and moral, which might be avoided; that means exist within our reach for their mitigation or removal; and that measures for prevention will effect infinitely more than remedies for the cure of disease."

If we compare the rate of mortality in New York fifty years ago, when it was 21 per 1000, with the present mortality, we shall find that from *want* of these improvements, there is a loss of 11,200 human lives in this city every year.—*Robbins on Sanitary Science.*

The average number of deaths by small-pox for the past ten years in this city, was 406.

| | | |
|---|---|---|
| The mortality of | London | is 25 in 1,000—13 more. |
| " | Berlin | is 25 in 1,000—13 " |
| " | Turin | is 26 in 1,000—12 " |
| " | Paris | is 28 in 1,000—10 " |
| " | Genoa | is 31 in 1,000— 7 " |
| " | Lyons | is 33 in 1,000— 5 " |
| " | Hamburgh | is 36 in 1,000— 2 " |
| " | New York | is 38 in 1,000— |

| | | | |
|---|---|---|---|
| The mortality | in Philadelphia, | in 1859, was | 1 in 63 |
| " | in Baltimore, | " | 1 in 50 |
| " | in Chicago, | " | 1 in 52 |
| " | in Providence, | " | 1 in 52 |
| " | in Boston, | " | 1 in 48 |
| " | in Brooklyn, | " | 1 in 40 |
| " | in New York, | " | 1 in 36 |

[*Report of Senate Committee.*

Reduce the death rate of New York to the death rate of Paris, and you will save 4,000 lives annually. Reduce the death rate of New York to the death rate of London, with a population thrice as great, and you will save more than 9,000 human lives every year. Make New York as healthy as it was fifty years ago, and you will save more than 11,000 human lives every year. And if you raise the health of New York to the standard proposed by the English General Board of Health, and the registrar-general, you will save annually nearly fifteen thousand lives. Therefore, basing our calculations on the practical and actual, we may without fear of the charge of exaggeration estimate that at least 10,000 people die unnecessary and preventable deaths in this city every year, chiefly from external filth and internal crowding and want of ventilation in their wretched habitations. *Are these lives worth saving?*—[*Robbins on Sanitary Science.*

One and a half per cent. per annum of the population is the standard rate of mortality in the most salubrious districts. Calculating from this datum, we have the following results for this city in 1860:

| Population | Deaths. | One in | Per centage. | Excess over 1½ per cent. |
|---|---|---|---|---|
| 814,277 | 22,710 | 35.85 | 2.78 | 10.496 |

If, therefore, the City of New York could last year, by any possibility, have been brought to a natural state of salubrity, its population this day would have been greater than it is by 10,496. There were in this city, in 1860, 146,944 unnecessary cases of sickness.

| | |
|---|---|
| Expenses of sickness, including medicines, nursing and medical expenses, at $20 each, | $2,938,880 00 |
| To loss of time, | 2,012,500 00 |
| Cost of unnecessary sickness in New York, in 1860, | $4,951,380 00 |

[*Griscom on Sanitary Legislation.*

